# Polymeric coordination complex of lithium(I) with aqua and cyanurate ligands

**DOI:** 10.1107/S2056989021009324

**Published:** 2021-09-14

**Authors:** Anjapuli Ponnuvel, Arumugam Pillai Kala, Karachalacherevu Seetharamiah Nagaraja, Chandran Karnan

**Affiliations:** a Department of Physics, Government Arts College for Men (Autonomous), University of Madras, Nandanam, Chennai 600 035, India; b Department of Chemistry, Dr. M.G.R. Educational and Research Institute, Chennai 600 095, India; c Department of Physics, Dr. M.G.R. Educational and Research Institute, Chennai 600 095, India

**Keywords:** cyanurate anion, five-coordinate Li^+^ cation, four-coordinate Li^+^ cation, bridging aqua ligand, supra­molecular assembly, hydrogen-bonding motifs, band gap, thermogravimetric analysis, crystal structure

## Abstract

The polymeric title complex, poly[hexa-μ-aqua-diaquatetra-μ-cyanurato-tetralithium] [Li_4_(C_3_H_2_N_3_O_3_)_4_(H_2_O)_7_]_*n*_, synthesized at room temperature from an aqueous solution of lithium hydroxide and cyanuric chloride, crystallizes in the triclinic space group *P* . There are two distinct Li^+^ cations in the asymmetric unit, one of which, Li1, has distorted trigonal–bipyramidal geometry and is coordinated *via* oxygen to two cyanurate anions occupying equatorial positions, and three water mol­ecules, two in the axial positions and the third in an equatorial position. One of the axial water ligands and the equatorial water ligand are involved in bridging to a crystallographically equivalent Li1 cation. A centre of inversion lies between the two Li1 cations and the Li1⋯Li1 distance is 3.037 (5) Å. The remaining axial water ligand bridges to the second Li cation, Li2, which is disordered over two crystallographic sites with approximately equal occupancy, and has an Li1⋯Li2 distance of 3.438 (7) Å. The terminal Li2 cation is coordinated to three water mol­ecules and an oxygen atom from a cyanuric anion and has a distorted tetra­hedral geometry. A three-dimensional network of inter­molecular hydrogen bonds involving N—H⋯O, O—H⋯O and O—H⋯N inter­actions serves to hold the structure together. The title compound was further characterized using IR and UV–vis spectroscopy and TG–DTA analysis.

## Abstract 2

The polymer contains Li^+^ cations coordinated *via* oxygen to two cyanuranate anions and three water mol­ecules in a trigonal–bipyramidal geometry and to three water mol­ecules and an oxygen from the cyanuric anion in a tetra­hedral geometry. A three-dimensional network of hydrogen bonds serves to hold the structure together.

## Chemical context   

A number of physical and structural properties, including mol­ecular geometry, metal–ligand bonding and directional supra­molecular architecture, control and influence the applications of hybrid metallo-organic coordination compounds (Coubrough *et al.*, 2019 ▸). Such compounds find potential applications in catalysis, gas storage, ion exchange, magnetic materials, sensors, optics and batteries (Qu *et al.*, 2016 ▸). The various possible metal and linker combinations are endless and have led to the synthesis of thousands of new materials with different metal geometries and functionalities (Chatenever *et al.*, 2019 ▸). Among the metals investigated, lithium-based complexes have unique advantages, exploiting properties of the lithium cation such as small ionic radius, high polarizing power, aqueous solubility and low economic cost (Ge *et al.*, 2018 ▸; Wan *et al.*, 2012 ▸). In solution, the lithium cation is of great importance because it can bind with selective organic ligands, leading to uses in many areas, including as active cellular components in ion-selective electrodes (ISE) in medicine, in nuclear power and in batteries (Ivanova *et al.*, 2019 ▸).

Cyanuric acid (1,3,5-triazine-2,4,6-triol) is an industrially important compound used to make pesticides, dyes, and disinfectants (Cho *et al.*, 2014 ▸). The acid is used as a chlorine stabilizer for outdoor swimming pools and sizeable industrial water systems. It is non-toxic to human and aqua­tic animals. It also has the remarkable property of biodegradability by soil bacteria (Prabhaharan *et al.*, 2015 ▸) and was recently found to be an effective nucleating agent during kinetic studies of biodegradable poly(l-lactide) and poly(3-hy­droxy­lbutyrate) co-polyesters (Pan *et al.*, 2013 ▸; Weng & Qiu, 2014 ▸).

**Figure 1:**
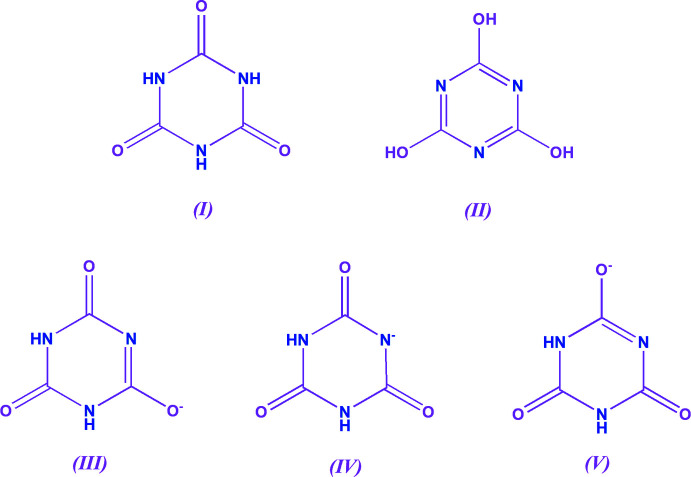
Tautomerism of cyanuric acid showing the trione (I) and triol (II) forms together with the resonance structures of the cyanurate anion [(III), (IV) and (V)].

With regard to metallo-organic chemistry, cyanuric acid is an important ligand due to its structural symmetry based on a planar six-membered ring, the existence of canonical structures and the presence of multiple hydrogen-bond-donor centres (Divya *et al.*, 2017 ▸). In its neutral, undissociated form, cyanuric acid shows tautomerism and can exist in the keto (I) or enol (II) forms (Fig. 1 ▸) (Abu-Salem *et al.*, 2017 ▸). In basic solution, it forms an anion with resonance between the (III), (IV) and (V) forms (Fig. 1 ▸).
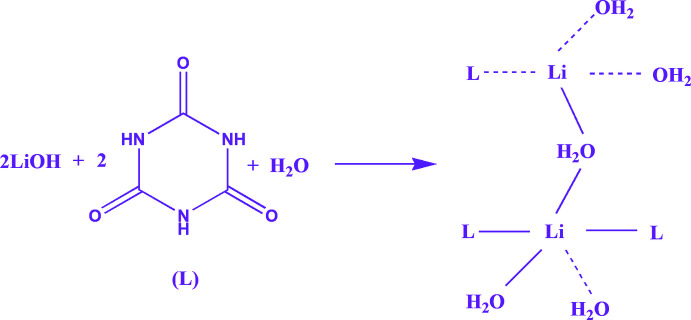


As cyanuric acid has three hydrogen-bonding-donor amine sites and three hydrogen-bonding-acceptor keto sites, it has been the subject of several structural and crystal-design studies (Shemchuk *et al.*, 2017 ▸). In the present work, we report the synthesis of a new lithium complex of cyanuric acid, [Li_4_(C_3_H_2_N_3_O_3_)_4_(H_2_O)_7_]_*n*_. The complex has been characterized by single-crystal X-ray diffraction, FTIR and UV–Vis spectroscopy, and TG–DTA analysis.

## Structural commentary   

**Figure 2:**
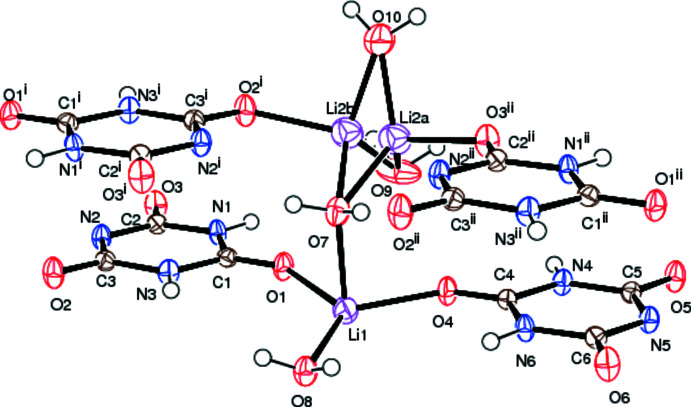
Coordination environments of the Li^+^ ions in the title compound with the displacement ellipsoids shown at the 50% probability level. Li2 is disordered over two sites, Li2*A* and Li2*B*, of approximately equal occupancy. [Symmetry codes: (i) −*x* + 2, −*y* + 1, −*z* + 1; (ii) −*x*, *y* + 1, *z*.]

The title compound crystallizes in the triclinic space group *P*ī. The asymmetric unit comprises two lithium ions, two cyan­urate ligands and three and a half coordinated water mol­ecules. An inversion centre lies between the related Li^+^ cations, Li1 and Li1^i^, generating a mol­ecular unit of formula [Li_4_(C_3_H_2_N_3_O_3_)_4_(H_2_O)_7_] (Fig. 2 ▸).

The two crystallographically distinct cyanurate ligands exist in resonance form (IV) (Fig. 1 ▸), in which the negative charge is located on a nitro­gen atom. Inter­estingly, for both ligands, coordination to lithium does not involve the deprotonated N2 and N5 atoms, but occurs *via* the keto oxygen atoms opposite (O1 and O4). This coordination preference may be due to the hard acid, Li^+^, preferring to bond to the harder base *i.e.* oxygen.

**Figure 3:**
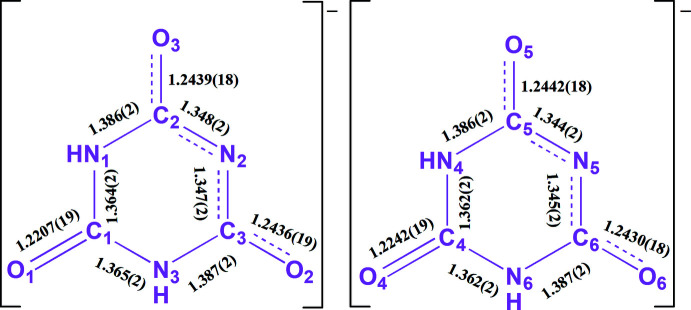
Observed bond lengths in the cyanurate anions found in the title compound. The delocalization of the negative charge on the deprotonated nitro­gen atoms (N2 and N5) over the adjacent keto groups is shown as dashed lines.

The C=O groups involved in coordination to Li1, namely C1=O1 and C4=O4 have bond lengths of 1.2207 (19) and 1.2242 (19) Å, respectively (Fig. 3 ▸), which are similar values to those found in related complexes (Divya *et al.*, 2020 ▸). The remaining two C=O groups in each ligand are involved in resonance and inter­molecular hydrogen bonding (and, in the cases of C2=O3 and C3=O2, in bonding to Li2*A* and Li2*B*) and have slightly longer bond lengths: C2—O3, 1.2439 (18) Å; C3—O2, 1.2436 (19) Å; C5—O5, 1.2442 (18) Å and C6—O6, 1.2430 (18) Å. The delocalization of the negative charge on the deprotonated nitro­gen atoms (N2 and N5) over the adjacent keto groups is shown as dashed lines in Fig. 2 ▸.

There are two distinct Li^+^ cations in the asymmetric unit (Fig. 3 ▸). Li1 has a distorted trigonal–bipyramidal geometry and is coordinated *via* O1 and O4 to the two cyanurate anions, which occupy equatorial positions, and three water mol­ecules, two (H_2_O7 and H_2_O8^i^) in the axial positions and the third (H_2_O8) in an equatorial position. The Li1—O bond lengths lie in the range 2.012 (3)– 2.201 (3) Å and the bond angles of O4—Li1—O1 = 118.40 (13)°, O4—Li1—O8 = 120.78 (14)°, O1—Li1—O8 = 120.74 (14)° and O8^i^—Li1—O7 = 178.56 (15)° confirm the trigonal–bipyramidal Li1 coordination geometry. One of the axial water ligands, H_2_O8^i^, and the equatorial water ligand, H_2_O7, bridge to a crystallographically equivalent Li1 cation. The Li1⋯Li1^i^ distance is 3.037 (5) Å, which is larger than the Li—Li bond distance found in lithium metal. The Li1—O—Li1^i^ bridge angle is 95.00 (11)°. The Li1—O8 and Li1—O8^i^ bond lengths are 2.032 (3) and 2.086 (3) Å, respectively.

The remaining axial water ligand, H_2_O7, bridges to the second Li^+^ cation, Li2, which is disordered over two sites, Li2*A* and Li2*B*, which have approximately equal occupancies. The Li1⋯Li2*A* and Li1⋯Li2*B* distances are 3.438 (7) and 3.439 (7) Å, respectively. Li2 is coordinated to two more water mol­ecules, H_2_O9, H_2_O10 and an oxygen atom from a cyanurate ligand (either O3^ii^ for Li2*A* or O2^iii^ for Li2*B*) to complete its distorted tetra­hedral coordination geometry. The Li2—O bond lengths lie in the range 1.931 (7)–2.057 (7) Å and the O—Li2—O angles in the range 97.9 (3)–125.3 (3)°

## Supra­molecular features   

**Figure 4:**
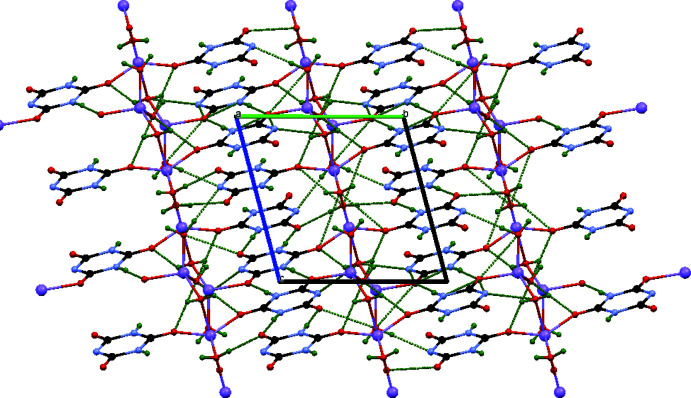
View of the crystal packing of the title compound along the *a* axis. Hydrogen-bonding inter­actions are indicated by green dashed lines.

Strong inter­molecular hydrogen-bonding inter­actions (Table 1 ▸) link the individual [Li_4_(C_3_H_2_N_3_O_3_)_4_(H_2_O)_7_] units into a three-dimensional network (Fig. 4 ▸). These involve inter­actions between water mol­ecule H_2_O8 and the adjacent cyanurate anions [O8⋯O6^iv^, O8⋯O5^ii^ at 2.7412 (16) and 2.7443 (16) Å, respectively].

In addition, each cyanurate moiety forms two strong hydrogen bonds between the N—H groups and oxygen atoms of adjacent mol­ecules with N⋯O distances in the range 2.7964 (16)–2.8054 (17) Å (N1—H1⋯O3^i^, N3—H3⋯O5^ii^, N4—H4⋯O6^iii^ and N6—H6⋯O6^iv^). Weaker hydrogen-bonding inter­actions, with N⋯O distances in the range 2.905 (2)–3.0342 (19) Å are also observed between the unprotonated N atoms of the cyanurate ions and nearby water mol­ecules (O7⋯N2^vii^, O7⋯N2^viii^ and O10⋯N5^v^, O10⋯N5^vi^).

Overall a supra­molecular hydrogen-bonded assembly is formed, as seen previously in other systems (Suguna *et al.*, 2014 ▸; Jeseentharani *et al.*, 2010 ▸).

## Database survey   

A survey of the Cambridge Structural Database (CSD version 5.42, May 2021 update; Groom *et al.*, 2016 ▸) revealed three polymeric metal complexes containing ligands related to the cyanurate ligand. These are (*μ*_2_-4,4′-bi­pyridine)­bis­[4,6-dihy­droxy-1,3,5-triazin-2(1*H*)-olato]dicopper(I) (WICCIV; Yue *et al.*, 2006 ▸) and *catena*-[bis­(*μ*-4,6-dioxo-1,4,5,6-tetra­hydro-1,3,5-triazin-2-olato)tetra­aqua­strontium(II)] (QEHKOG; Divya *et al.*, 2017 ▸), both of which crystallize in the monoclinic crystal system, together with *catena*-[tetra­kis­(*μ*-2,4,6-trioxo-1,3,5-triazinan-1-ide)bis­(*μ*-aqua)­tetra­aqua­copper(II)disodium(I)] (KUXFAK02; Divya *et al.*, 2020 ▸), which, like the title compound, crystallizes in the triclinic space group *P*ī.

## Fourier transform infrared spectroscopy   

**Figure 5:**
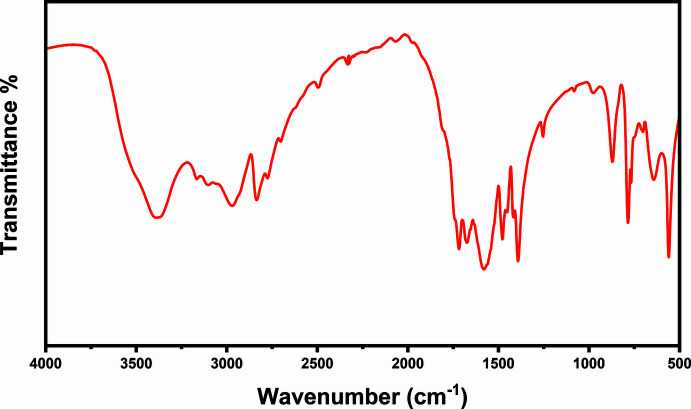
The infrared spectrum of the title compound.

The FTIR spectrum of the title compound was measured using a Perkin Elmer Spectrum One instrument over a 450–4000 cm^−1^ scan range at 1.0 cm^−1^ resolution (Fig. 5 ▸). The bands at 3400 (*sh*, *m*) and 3389 cm^−1^ (*br*) correspond to ν(O—H) (Prabhaharan *et al.*, 2015 ▸; Bourzami *et al.*, 2018 ▸) and those at 3165, 3102 and 2831 cm^−1^ to ν(N—H) (Divya *et al.*, 2020 ▸; Surinwong *et al.*, 2014 ▸). The bands at 1718 and 1675 cm^−1^ correspond to ν(C=O) (Divya *et al.*, 2020 ▸; Vu *et al.*, 2019 ▸) and those at 1578 and 1478 cm^−1^ to ν_sym_(C—N) (Surinwong *et al.*, 2014 ▸). The wavenumbers of the vibrations involving the N—H, C=N and C=O groups are affected by the partial delocal­ization of electron charge density on one part of the ring, as shown in Fig. 2 ▸, and by the to coordination of C=O oxygen to Li^+^. Finally the bands at 870, 784 and 559 cm^−1^ are attributed to the characteristic vibrations of the 1,3,5-triazine ring (Bourzami *et al.*, 2018 ▸; Bellardita *et al.*, 2018 ▸).

## Absorption spectroscopy   

**Figure 6:**
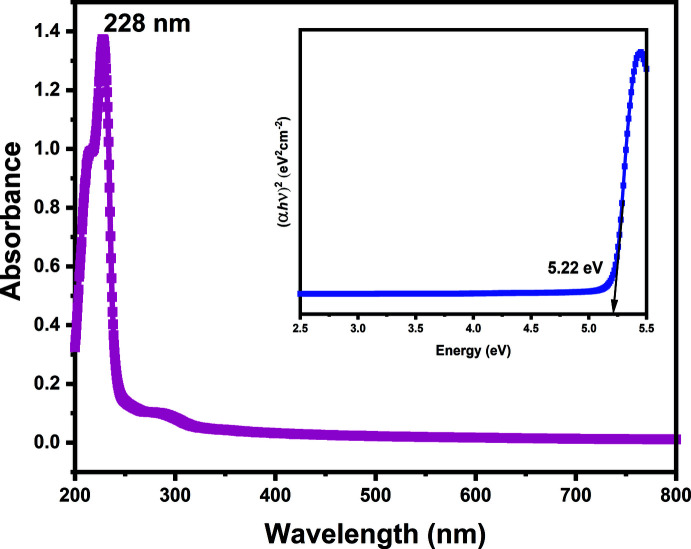
The absorption spectrum of the title compound. The direct bandgap, *E*_g_, is estimated from the plot in the inset to be 5.22 eV.

**Figure 7:**
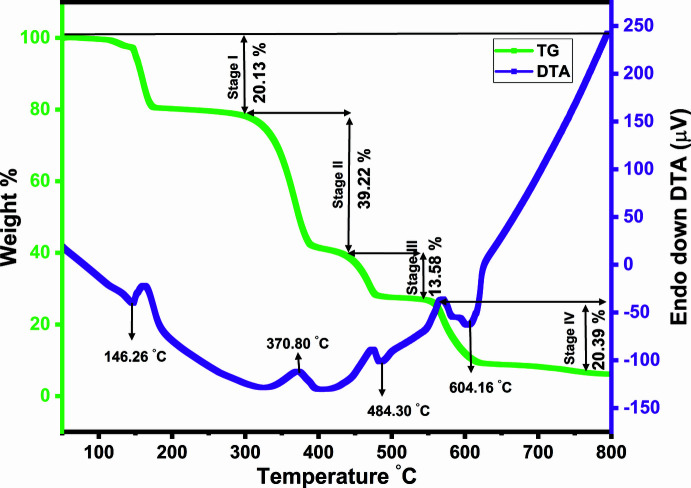
TG–DTA of the title compound measured under an N_2_ atmosphere using a heating rate of 10°C min^−1^.

The UV–Vis NIR absorption spectrum was measured using a Perkin Elmer lambda 950 UV–Vis–NIR spectrophotometer (Fig. 6 ▸). The peaks observed at 290 and 228 nm are due to π–π* and n–π* transitions, respectively (Qiu & Gao, 2005 ▸; Moreno-Guerra *et al.*, 2019 ▸). The band gap, *E*_g_, can be estimated from the maximum absorption at 228 nm using the following equations. The optical absorption coefficient, α, is related to the absorbance, *A*, by the relations: α = 2.303 *A*/*t* and α = *A*(*h*ν - *E*_g_)^1/2^/hν, where *t* is the thickness of the crystal (1 mm) and hν is the photon energy. A plot of (α*h*ν)^2^ *versus h*ν is shown as the inset in Fig. 7 ▸, from which the band gap (*E*_g_) is estimated to be 5.22 eV.

## Thermogravimetric and differential thermal analysis   

Simultaneous TG–DTA measurements and analysis of weight change and heat flow were performed using a Perkin Elmer STA 6000 instrument operating at a scanning rate of 10°C min^−1^ with a resolution of 1 µg under a dry N_2_ atmosphere. The thermogram (Fig. 7 ▸) shows four stages of decomposition. The first stage starts at 92°C and ends at 172°C with a deriv­ative peak at 146.26°C and a measured weight loss of 20.13%, which is in reasonable agreement with the loss of the seven coordinated water mol­ecules (calculated weight loss 18.91%). The second and third stages of decomposition, occurring from 298 to 550°C, correspond to the decomposition of the cyanurate ligands with a measured total weight loss of 52.80%, leading to the formation of LiNO_3_ (calculated weight loss 51.07%) (Divya *et al.*, 2020 ▸). In the fourth decomposition stage, occurring from 550 to 662°C, LiNO_3_ decomposes with a measured weight loss of 20.39% to produce Li_2_O as the final solid residue (calculated weight loss 21.67%).

## Synthesis and crystallization   

Lithium hydroxide (1.25 g, 0.052 mol; LOBA) and cyanuric chloride (1.84 g, 0.01 mol; Sigma–Aldrich) were dissolved in water (100 ml). The resulting solution was stirred for 5 h at ambient temperature (300-301 K) and filtered twice using Whatman filter paper. The solvent was allowed to evaporate in a dust-free environment. After 22 days, good quality colourless crystals were harvested.

## Refinement   

Crystal data, data collection and structure refinement details are summarized in Table 2 ▸. Li2 was found to be disordered over two positions, Li2*A* and Li2*B*, which were resolved using the PART command (Sheldrick, 2015*b* ▸) with an occupancy ratio of 0.501 (6):0.499 (6). The N-bound H atoms were placed geometrically and refined using a riding model with respect to their parent atoms using AFIX 43 with N—H = 0.86 Å and *U*_iso_(H) = 1.2*U*_eq_(N). The hydrogen atoms on the water mol­ecules were located in difference-Fourier maps and each *U*_iso_(H) parameter was freely refined with the O—H distance restrained to 0.85 (2) Å using DFIX. The H—O—H angle distances were restrained using DFIX to a target value of 1.39 (2) Å [or 1.41 (2) Å for H9*A*—O9—H9*B*] in order to keep the water mol­ecules close to their standard geometries.

## Supplementary Material

Crystal structure: contains datablock(s) I. DOI: 10.1107/S2056989021009324/cq2044sup1.cif

Structure factors: contains datablock(s) I. DOI: 10.1107/S2056989021009324/cq2044Isup2.hkl

CCDC reference: 1991191

Additional supporting information:  crystallographic information; 3D view; checkCIF report

## Figures and Tables

**Table 1:** Hydrogen-bond geometry (Å, °)

*D*—H⋯*A*	*D*—H	H⋯*A*	*D*⋯*A*	*D*—H⋯*A*
N1—H1⋯O3^i^	0.86	1.95	2.8054 (17)	175
N3—H3⋯O5^ii^	0.86	1.94	2.7964 (16)	173
N4—H4⋯O2^iii^	0.86	1.95	2.8033 (17)	175
N6—H6⋯O6^iv^	0.86	1.95	2.8002 (17)	172
O8—H8*A*⋯O6^iv^	0.86 (2)	1.92 (2)	2.7412 (16)	159 (2)
O8—H8*B*⋯O5^ii^	0.86 (2)	1.92 (2)	2.7443 (16)	160 (2)
O10—H10*A*⋯N5^v^	0.87 (2)	2.16 (2)	3.0311 (19)	177 (3)
O10—H10*B*⋯N5^vi^	0.86 (2)	2.18 (2)	3.0342 (19)	174 (3)
O7—H7*A*⋯N2^vii^	0.88 (3)	2.09 (3)	2.908 (2)	154 (2)
O7—H7*B*⋯N2^viii^	0.88 (2)	2.09 (2)	2.905 (2)	153 (2)

Symmetry codes: (i) -x+1, -y, -z+2; (ii) x+1, y-1, z; (iii) x-1, y+1, z; (iv) -x+2, -y+2, -z+1; (v) -x+1, -y+2, -z+2; (vi) x, y-1, z+1; (vii) x, y+1, z; (viii) -x+2, -y, -z+2.

**Table 2:** Experimental details

Crystal data	
Chemical formula	[Li_4_(C_3_H_2_N_3_O_3_)_4_(H_2_O)_7_]
*M*_r_	666.17
Crystal system, space group	Triclinic, *P*\overline{1}
Temperature (K)	296
*a*, *b*, *c* (Å)	8.8530 (5), 9.0592 (6), 9.6621 (6)
α, β, γ (°)	67.806 (2), 62.887 (2), 68.580 (2)
*V* (Å^3^)	620.89 (7)
*Z*	1
Radiation type	Mo *K*α
μ (mm^−1^)	0.16
Crystal size (mm)	0.15 × 0.15 × 0.10

Data collection	
Diffractometer	Bruker Kappa *APEX3* CMOS
Absorption correction	Multi-scan (*SADABS*; Bruker, 2016 ▸)
*T*_min_, *T*_max_	0.707, 0.746
No. of measured, independent and observed [*I* > 2σ(*I*)] reflections	17961, 2181, 1917
*R*_int_	0.027
(sin θ/λ)_max_ (Å^−1^)	0.594

Refinement	
*R*[*F*^2^ > 2σ(*F* ^2^)], *wR*(*F* ^2^), *S*	0.039, 0.115, 1.14
No. of reflections	2181
No. of parameters	250
No. of restraints	12
H-atom treatment	H atoms treated by a mixture of independent and constrained refinement
Δρ_max_, Δρ_min_ (e Å^−3^)	0.38, −0.32

Computer programs: *APEX3*, *SAINT*/*XPREP* (Bruker, 2016 ▸), *SHELXT2014/5* (Sheldrick, 2015*a* ▸), *SHELXL2018/3* (Sheldrick, 2015*b* ▸), *ORTEP-3 for Windows* (Farrugia, 2012 ▸) and *Mercury* (Macrae *et al.*, 2020 ▸).

## supplementary crystallographic information

### Crystal data

**Table:**

[Li_4_(C_3_H_2_N_3_O_3_)_4_(H_2_O)_7_]	*Z* = 1
*M**_r_* = 666.17	*F*(000) = 342
Triclinic, *P*1	*D*_x_ = 1.782 Mg m^−^^3^
*a* = 8.8530 (5) Å	Mo *K*α radiation, λ = 0.71073 Å
*b* = 9.0592 (6) Å	Cell parameters from 9866 reflections
*c* = 9.6621 (6) Å	θ = 3.2–30.5°
α = 67.806 (2)°	µ = 0.16 mm^−^^1^
β = 62.887 (2)°	*T* = 296 K
γ = 68.580 (2)°	Block, colourless
*V* = 620.89 (7) Å^3^	0.15 × 0.15 × 0.10 mm

### Data collection

**Table:**

Bruker Kappa APEX3 CMOS diffractometer	2181 independent reflections
Radiation source: fine-focus sealed tube	1917 reflections with *I* > 2σ(*I*)
Graphite monochromator	*R*_int_ = 0.027
ω and φ scan	θ_max_ = 25.0°, θ_min_ = 2.9°
Absorption correction: multi-scan (SADABS; Bruker, 2016)	*h* = −10→10
*T*_min_ = 0.707, *T*_max_ = 0.746	*k* = −10→10
17961 measured reflections	*l* = −11→11

### Refinement

**Table:**

Refinement on *F*^2^	12 restraints
Least-squares matrix: full	Hydrogen site location: mixed
*R*[*F*^2^ > 2σ(*F*^2^)] = 0.039	H atoms treated by a mixture of independent and constrained refinement
*wR*(*F*^2^) = 0.115	*w* = 1/[σ^2^(*F*_o_^2^) + (0.0656*P*)^2^ + 0.1885*P*] where *P* = (*F*_o_^2^ + 2*F*_c_^2^)/3
*S* = 1.14	(Δ/σ)_max_ < 0.001
2181 reflections	Δρ_max_ = 0.38 e Å^−^^3^
250 parameters	Δρ_min_ = −0.31 e Å^−^^3^

### Special details

**Table:**

Geometry. All esds (except the esd in the dihedral angle between two l.s. planes) are estimated using the full covariance matrix. The cell esds are taken into account individually in the estimation of esds in distances, angles and torsion angles; correlations between esds in cell parameters are only used when they are defined by crystal symmetry. An approximate (isotropic) treatment of cell esds is used for estimating esds involving l.s. planes.

### Fractional atomic coordinates and isotropic or equivalent isotropic displacement parameters (Å^2^)

**Table:**

	*x*	*y*	*z*	*U*_iso_*/*U*_eq_	Occ. (<1)
Li1	0.8785 (3)	0.4997 (3)	0.6728 (3)	0.0273 (6)	
Li2A	0.6581 (8)	0.5613 (9)	1.0527 (8)	0.0372 (10)	0.501 (6)
Li2B	0.6884 (8)	0.4388 (9)	1.0835 (8)	0.0372 (10)	0.499 (6)
C1	0.84110 (19)	0.13728 (18)	0.83772 (17)	0.0162 (3)	
C2	0.75963 (19)	−0.12382 (18)	0.95319 (17)	0.0167 (3)	
C3	1.05252 (19)	−0.12405 (18)	0.85818 (17)	0.0172 (3)	
C4	0.65967 (18)	0.86155 (18)	0.65700 (17)	0.0159 (3)	
C5	0.44821 (19)	1.12286 (18)	0.64435 (17)	0.0165 (3)	
C6	0.74036 (19)	1.12290 (18)	0.54318 (17)	0.0167 (3)	
N1	0.71916 (16)	0.04611 (15)	0.91183 (15)	0.0186 (3)	
H1	0.611046	0.096942	0.934161	0.022*	
N2	0.92688 (16)	−0.20772 (15)	0.92701 (15)	0.0196 (3)	
N3	1.00835 (16)	0.04588 (16)	0.81138 (15)	0.0190 (3)	
H3	1.091204	0.096662	0.762906	0.023*	
N4	0.49252 (16)	0.95287 (15)	0.68709 (15)	0.0186 (3)	
H4	0.409623	0.902156	0.735402	0.022*	
N5	0.57311 (16)	1.20723 (15)	0.57313 (15)	0.0187 (3)	
N6	0.78129 (15)	0.95284 (15)	0.58268 (15)	0.0186 (3)	
H6	0.889544	0.902188	0.558918	0.022*	
O1	0.80308 (14)	0.28696 (13)	0.79896 (13)	0.0236 (3)	
O2	1.20853 (13)	−0.19384 (13)	0.83558 (14)	0.0255 (3)	
O3	0.63820 (13)	−0.19370 (13)	1.01218 (14)	0.0250 (3)	
O4	0.69679 (14)	0.71143 (13)	0.69329 (13)	0.0232 (3)	
O5	0.29078 (13)	1.19154 (13)	0.67448 (14)	0.0247 (3)	
O6	0.86314 (13)	1.19169 (13)	0.48001 (14)	0.0254 (3)	
O7	0.89868 (15)	0.49969 (14)	0.89151 (14)	0.0262 (3)	
O8	1.13401 (15)	0.50005 (14)	0.53775 (14)	0.0247 (3)	
O9	0.500000	0.500000	1.000000	0.0469 (6)	
O10	0.5956 (2)	0.50030 (18)	1.28285 (17)	0.0489 (4)	
H7A	0.939 (3)	0.582 (2)	0.877 (3)	0.064 (8)*	
H7B	0.981 (3)	0.415 (2)	0.915 (3)	0.059 (7)*	
H8A	1.158 (3)	0.5834 (19)	0.538 (3)	0.039 (6)*	
H8B	1.201 (3)	0.415 (2)	0.577 (3)	0.047 (6)*	
H9A	0.433 (6)	0.584 (4)	1.047 (5)	0.057 (15)*	0.5
H9B	0.472 (6)	0.418 (4)	1.089 (4)	0.043 (13)*	0.5
H10A	0.543 (4)	0.585 (2)	1.323 (3)	0.069 (8)*	
H10B	0.581 (3)	0.418 (2)	1.365 (2)	0.062 (8)*	

### Atomic displacement parameters (Å^2^)

**Table:**

	*U*^11^	*U*^22^	*U*^33^	*U*^12^	*U*^13^	*U*^23^
Li1	0.0251 (13)	0.0217 (14)	0.0277 (13)	−0.0063 (11)	−0.0042 (11)	−0.0048 (11)
Li2A	0.033 (2)	0.039 (2)	0.035 (2)	−0.010 (2)	−0.0070 (18)	−0.010 (2)
Li2B	0.033 (2)	0.039 (2)	0.035 (2)	−0.010 (2)	−0.0070 (18)	−0.010 (2)
C1	0.0177 (7)	0.0159 (8)	0.0150 (7)	−0.0045 (6)	−0.0050 (6)	−0.0045 (6)
C2	0.0165 (7)	0.0161 (8)	0.0172 (7)	−0.0049 (6)	−0.0052 (6)	−0.0043 (6)
C3	0.0157 (7)	0.0173 (8)	0.0169 (7)	−0.0037 (6)	−0.0048 (6)	−0.0042 (6)
C4	0.0168 (7)	0.0145 (8)	0.0154 (7)	−0.0020 (6)	−0.0064 (6)	−0.0039 (6)
C5	0.0162 (7)	0.0156 (8)	0.0165 (7)	−0.0026 (6)	−0.0065 (6)	−0.0034 (6)
C6	0.0160 (7)	0.0164 (8)	0.0174 (7)	−0.0041 (6)	−0.0051 (6)	−0.0052 (6)
N1	0.0122 (6)	0.0141 (7)	0.0268 (7)	−0.0016 (5)	−0.0059 (5)	−0.0056 (5)
N2	0.0157 (7)	0.0144 (7)	0.0263 (7)	−0.0036 (5)	−0.0071 (5)	−0.0033 (5)
N3	0.0143 (6)	0.0152 (7)	0.0246 (7)	−0.0068 (5)	−0.0047 (5)	−0.0020 (5)
N4	0.0134 (6)	0.0140 (7)	0.0248 (7)	−0.0050 (5)	−0.0054 (5)	−0.0017 (5)
N5	0.0163 (7)	0.0135 (7)	0.0249 (7)	−0.0035 (5)	−0.0078 (5)	−0.0034 (5)
N6	0.0112 (6)	0.0143 (7)	0.0266 (7)	−0.0010 (5)	−0.0052 (5)	−0.0054 (5)
O1	0.0246 (6)	0.0127 (6)	0.0303 (6)	−0.0043 (5)	−0.0097 (5)	−0.0029 (5)
O2	0.0128 (5)	0.0211 (6)	0.0370 (7)	−0.0023 (4)	−0.0075 (5)	−0.0054 (5)
O3	0.0156 (6)	0.0189 (6)	0.0372 (7)	−0.0076 (5)	−0.0064 (5)	−0.0047 (5)
O4	0.0219 (6)	0.0127 (6)	0.0307 (6)	−0.0019 (5)	−0.0089 (5)	−0.0041 (5)
O5	0.0129 (5)	0.0177 (6)	0.0370 (7)	−0.0014 (4)	−0.0082 (5)	−0.0038 (5)
O6	0.0156 (6)	0.0189 (6)	0.0393 (7)	−0.0066 (5)	−0.0070 (5)	−0.0064 (5)
O7	0.0294 (6)	0.0196 (6)	0.0330 (7)	−0.0040 (5)	−0.0164 (5)	−0.0056 (5)
O8	0.0237 (6)	0.0185 (6)	0.0313 (6)	−0.0047 (5)	−0.0108 (5)	−0.0049 (5)
O9	0.0230 (10)	0.0917 (18)	0.0275 (10)	−0.0221 (11)	0.0023 (8)	−0.0229 (12)
O10	0.0759 (11)	0.0278 (8)	0.0380 (8)	−0.0076 (7)	−0.0217 (8)	−0.0066 (6)

### Geometric parameters (Å, º)

**Table:**

Li1—O4	2.012 (3)	C3—N3	1.387 (2)
Li1—O1	2.017 (3)	C4—O4	1.2242 (19)
Li1—O8	2.032 (3)	C4—N4	1.362 (2)
Li1—O8^i^	2.086 (3)	C4—N6	1.362 (2)
Li1—O7	2.201 (3)	C5—O5	1.2442 (18)
Li1—Li1^i^	3.037 (5)	C5—N5	1.344 (2)
Li1—Li2A	3.438 (7)	C5—N4	1.386 (2)
Li1—Li2B	3.439 (7)	C6—O6	1.2430 (18)
Li2A—O10	1.931 (7)	C6—N5	1.345 (2)
Li2A—O9	1.989 (6)	C6—N6	1.387 (2)
Li2A—O7	2.010 (6)	N1—H1	0.8600
Li2A—O3^ii^	2.057 (7)	N3—H3	0.8600
Li2B—O10	1.931 (7)	N4—H4	0.8600
Li2B—O9	1.988 (7)	N6—H6	0.8600
Li2B—O7	2.010 (6)	O7—H7A	0.880 (16)
Li2B—O2^iii^	2.056 (7)	O7—H7B	0.883 (16)
C1—O1	1.2207 (19)	O8—H8A	0.857 (15)
C1—N1	1.364 (2)	O8—H8B	0.862 (15)
C1—N3	1.365 (2)	O9—H9A	0.905 (19)
C2—O3	1.2439 (18)	O9—H9B	0.898 (19)
C2—N2	1.348 (2)	O9—H9A^iv^	0.905 (19)
C2—N1	1.386 (2)	O9—H9B^iv^	0.898 (19)
C3—O2	1.2436 (19)	O10—H10A	0.872 (16)
C3—N2	1.347 (2)	O10—H10B	0.859 (16)
			
O4—Li1—O1	118.40 (13)	O4—C4—N6	123.28 (13)
O4—Li1—O8	120.78 (14)	N4—C4—N6	113.72 (13)
O1—Li1—O8	120.74 (14)	O5—C5—N5	122.46 (14)
O4—Li1—O8^i^	93.98 (13)	O5—C5—N4	117.42 (13)
O1—Li1—O8^i^	94.00 (13)	N5—C5—N4	120.13 (13)
O8—Li1—O8^i^	85.00 (11)	O6—C6—N5	122.42 (14)
O4—Li1—O7	86.70 (11)	O6—C6—N6	117.45 (13)
O1—Li1—O7	86.78 (11)	N5—C6—N6	120.13 (13)
O8—Li1—O7	93.57 (12)	C1—N1—C2	123.71 (13)
O8^i^—Li1—O7	178.56 (15)	C1—N1—H1	118.1
O10—Li2A—O9	109.9 (3)	C2—N1—H1	118.1
O10—Li2A—O7	125.3 (3)	C3—N2—C2	119.06 (13)
O9—Li2A—O7	105.8 (3)	C1—N3—C3	123.85 (12)
O10—Li2A—O3^ii^	97.9 (3)	C1—N3—H3	118.1
O9—Li2A—O3^ii^	118.3 (3)	C3—N3—H3	118.1
O7—Li2A—O3^ii^	100.0 (3)	C4—N4—C5	123.81 (12)
O10—Li2B—O9	109.9 (3)	C4—N4—H4	118.1
O10—Li2B—O7	125.3 (3)	C5—N4—H4	118.1
O9—Li2B—O7	105.9 (3)	C5—N5—C6	118.47 (13)
O10—Li2B—O2^iii^	98.3 (3)	C4—N6—C6	123.70 (12)
O9—Li2B—O2^iii^	118.3 (3)	C4—N6—H6	118.2
O7—Li2B—O2^iii^	99.6 (3)	C6—N6—H6	118.2
O1—C1—N1	122.71 (13)	C1—O1—Li1	149.18 (13)
O1—C1—N3	123.42 (13)	C3—O2—Li2B^iii^	127.6 (2)
N1—C1—N3	113.86 (13)	C2—O3—Li2A^v^	127.3 (2)
O3—C2—N2	122.20 (14)	C4—O4—Li1	149.09 (13)
O3—C2—N1	117.99 (13)	Li2A—O7—Li1	109.4 (2)
N2—C2—N1	119.80 (13)	Li2B—O7—Li1	109.4 (2)
O2—C3—N2	122.28 (14)	Li1—O8—Li1^i^	95.00 (11)
O2—C3—N3	118.11 (13)	H8A—O8—H8B	106.4 (17)
N2—C3—N3	119.61 (13)	Li2A^iv^—O9—Li2A	180.0
O4—C4—N4	123.00 (13)	H10A—O10—H10B	104.5 (19)
			
O1—C1—N1—C2	178.28 (13)	O5—C5—N5—C6	−179.15 (14)
N3—C1—N1—C2	−1.8 (2)	N4—C5—N5—C6	0.8 (2)
O3—C2—N1—C1	−176.02 (13)	O6—C6—N5—C5	−178.87 (13)
N2—C2—N1—C1	3.1 (2)	N6—C6—N5—C5	1.2 (2)
O2—C3—N2—C2	177.57 (14)	O4—C4—N6—C6	−179.04 (13)
N3—C3—N2—C2	−1.9 (2)	N4—C4—N6—C6	1.1 (2)
O3—C2—N2—C3	177.99 (13)	O6—C6—N6—C4	177.82 (13)
N1—C2—N2—C3	−1.1 (2)	N5—C6—N6—C4	−2.3 (2)
O1—C1—N3—C3	178.49 (13)	N1—C1—O1—Li1	−166.0 (2)
N1—C1—N3—C3	−1.5 (2)	N3—C1—O1—Li1	14.1 (3)
O2—C3—N3—C1	−176.14 (13)	N2—C3—O2—Li2B^iii^	−8.1 (3)
N2—C3—N3—C1	3.4 (2)	N3—C3—O2—Li2B^iii^	171.4 (2)
O4—C4—N4—C5	−178.82 (13)	N2—C2—O3—Li2A^v^	−9.5 (3)
N6—C4—N4—C5	1.1 (2)	N1—C2—O3—Li2A^v^	169.6 (2)
O5—C5—N4—C4	177.88 (13)	N4—C4—O4—Li1	166.5 (2)
N5—C5—N4—C4	−2.1 (2)	N6—C4—O4—Li1	−13.4 (3)

Symmetry codes: (i) −*x*+2, −*y*+1, −*z*+1; (ii) *x*, *y*+1, *z*; (iii) −*x*+2, −*y*, −*z*+2; (iv) −*x*+1, −*y*+1, −*z*+2; (v) *x*, *y*−1, *z*.

### Hydrogen-bond geometry (Å, º)

**Table:**

*D*—H···*A*	*D*—H	H···*A*	*D*···*A*	*D*—H···*A*
N1—H1···O3^vi^	0.86	1.95	2.8054 (17)	175
N3—H3···O5^vii^	0.86	1.94	2.7964 (16)	173
N4—H4···O2^viii^	0.86	1.95	2.8033 (17)	175
N6—H6···O6^ix^	0.86	1.95	2.8002 (17)	172
O8—H8*A*···O6^ix^	0.86 (2)	1.92 (2)	2.7412 (16)	159 (2)
O8—H8*B*···O5^vii^	0.86 (2)	1.92 (2)	2.7443 (16)	160 (2)
O10—H10*A*···N5^x^	0.87 (2)	2.16 (2)	3.0311 (19)	177 (3)
O10—H10*B*···N5^xi^	0.86 (2)	2.18 (2)	3.0342 (19)	174 (3)
O7—H7*A*···N2^ii^	0.88 (3)	2.09 (3)	2.908 (2)	154 (2)
O7—H7*B*···N2^iii^	0.88 (2)	2.09 (2)	2.905 (2)	153 (2)

Symmetry codes: (ii) *x*, *y*+1, *z*; (iii) −*x*+2, −*y*, −*z*+2; (vi) −*x*+1, −*y*, −*z*+2; (vii) *x*+1, *y*−1, *z*; (viii) *x*−1, *y*+1, *z*; (ix) −*x*+2, −*y*+2, −*z*+1; (x) −*x*+1, −*y*+2, −*z*+2; (xi) *x*, *y*−1, *z*+1.
